# Toxicological Assessment of Pure Lolitrem B and Ryegrass Seed Infected with the AR37 Endophyte Using Mice

**DOI:** 10.3390/jof8111139

**Published:** 2022-10-28

**Authors:** Sarah C. Finch, Allan D. Hawkes, Joan M. Fitzgerald, Ric B. Broadhurst, Maryann R. Staincliffe, John S. Munday

**Affiliations:** 1 AgResearch Limited, Ruakura Research Centre, Private Bag 3123, Hamilton 3240, New Zealand; 2 School of Veterinary Science, Massey University, Private Bag 11 222, Palmerston North 4442, New Zealand

**Keywords:** feeding study, reproduction study, epoxyjanthitrems, lolitrem B, toxicology

## Abstract

Fungal endophytes in perennial ryegrass are essential to New Zealand’s pastoral system due to anti-insect effects. However, endophytes also produce compounds which can be detrimental to animals. Furthermore, as these toxins have been detected in the milk and fat of animals grazing common-toxic (containing lolitrem B) or AR37 endophyte-infected herbage they could enter the human food chain. To assess the risk to human health mice were fed for 90 days with three dose rates of lolitrem B and of AR37. Parameters indicative of animal health were measured as well as chemical, hematological and histological analysis of samples collected on day 90. Since endophyte toxin residues have been detected in milk, they could be transferred from mother to offspring via breast milk. To evaluate possible effects on reproduction two complete generations of mice were fed lolitrem B or AR37. At the dose rates given no adverse effects were observed in either study. The 100-fold safety factor to allow the use of animal data in human health assessments was applied and by considering the concentrations of lolitrem B or AR37 metabolites which could be ingested by a consumer it is highly unlikely that they pose any risk to human health.

## 1. Introduction

Perennial ryegrass (*Lolium perenne* L.) is the dominant pasture grass in New Zealand and is characterized by the presence of an endophyte (*Epichloë* var *lolii* (formerly *Neotyphodium*, formerly *Acremonium*) [1] to form a mutualistic association. The presence of an endophyte in perennial ryegrass was first recognized in 1935 [2] and is thought to have been unwittingly introduced through the importation of seed by early settlers [3]. In New Zealand, due to the environmental conditions, the presence of an endophyte in ryegrass is essential for plant productivity and persistence. This effect is largely driven by peramine [4], a secondary metabolite produced by the endophyte. This compound confers resistance to the key pasture pest, Argentine stem weevil (*Listronotus bonariensis*) [5] and has not been associated with any adverse effects in animals [6,7]. However, in addition to this beneficial metabolite, the naturally occurring (common-toxic) endophyte also produces compounds which induce animal disease. One of these, lolitrem B [8] is the cause of ryegrass staggers, a neurological disease of grazing animals, responsible for farm management issues as well as major production losses [9]. Another metabolite, ergovaline, causes reduced feed intake, an increase in body temperature and respiration, reproductive issues, fescue foot (a vascular necrosis of the extremities) and an overall loss of grazing animal production [10]. However, in addition to these negative effects, ergovaline does offer some protection against African black beetle (*Heteronychus arator*) adults [11].

To minimize the incidence of animal disease, while retaining anti-insect properties, a worldwide search for endophytes which have a favorable chemical profile has been conducted. Such an endophyte would produce metabolites responsible for anti-insect activity but no metabolites that cause animal disease. Once identified, such endophytes can then be artificially inoculated into modern grass cultivars [12] resulting in superior endophyte-grass associations. This approach has been very successful with a number of endophytic products now commercially available [3]. One such product, the AR37 endophyte, has proved to be very successful and will contribute NZ$3.6 billion to the New Zealand economy over the 20-year lifetime of its patent [13]. AR37 does not produce lolitrem B, ergovaline or peramine but does show activity against a broad range of insect pests including Argentine stem weevil larvae [14], adult African black beetle [15], porina (*Wiseana cervinata*) [16,17,18], root aphid [17,19,20] and pasture mealybug (*Balanococcus poae*) [21]. Furthermore, although AR37 does not express lolitrem B, it does induce ryegrass staggers although cases in animals grazing AR37 tend to be less frequent and less severe than those observed in animals grazing the common-toxic endophyte [22,23].

The fact that AR37 confers beneficial effects against insects and induces ryegrass staggers in animals is surprising given that this endophyte does not produce either peramine or lolitrem B. Further research identified the presence of five epoxyjanthitrems which were difficult to isolate due to their instability [24]. Due to the structural similarity to janthitrems, which are known to be tremorgenic [25], it was hypothesized that the epoxyjanthitrems could be responsible for the ryegrass staggers observed in animals grazing AR37. The testing of pure epoxyjanthitrem I, using an established mouse model for tremorgenicity [24], confirmed this to be the case. The pure compound was also shown to have potent activity against porina [24]. The lesson learnt from the development of AR37 is that endophytes express a myriad of compounds, some of which will be known but some, such as the epoxyjanthitrems in AR37, will be unknown. It is therefore difficult to predict the impact of a novel endophyte-grass association based on known chemistry alone and hence rigorous field testing is required.

With the commercial release of novel endophytes, the possible impact on food safety should be considered. Animals have been grazing the common-toxic endophyte since New Zealand was colonized and, as such, the population has been consuming products derived from these animals seemingly without any adverse effects. However, with new endophytes, new secondary metabolites will be consumed by animals giving rise to the possibility of them subsequently being consumed by humans. A risk assessment requires two pieces of information; how much of the contaminant could a person eat and how toxic is that contaminant. Previous studies have shown that cows fed common-toxic or AR37 endophyte-infected herbage produce milk containing lolitrem B (maximum of 5.0 ppb) or epoxyjanthitrems (maximum of 109.0 ppb), respectively [26]. Furthermore, fat biopsies taken regularly from sheep grazing perennial ryegrass with the common-toxic or the AR37 endophyte over the period when ryegrass staggers is typically observed showed lolitrem B (maximum of 61.8 ppb) and epoxyjanthitrems (maximum of 1032 ppb) were present, respectively [27]. In both studies the endophyte toxin residues did not accumulate but rather responded quickly to the amounts ingested by the animal. The presence of lolitrem B in fat is consistent with the work of Miyazaki et al. [28] who detected the toxin in the body fat of beef cattle exposed to the compound. These findings are not surprising given these toxins are highly lipophilic [29]. Although no information is known about the metabolism of the epoxyjanthitrems, some work on lolitrem B has been published [30].

In all of the published studies, the concentrations of the known toxins in animal products were low and it could be predicted that they would not cause an issue for human health. However, only known compounds can be screened for and as was seen in the early development of AR37, the profile of secondary metabolites can be radically altered. Therefore, it is highly likely that many more unknown compounds are produced by the AR37 endophyte and hence could be present in human food products. To ensure that both known and unknown compounds are included in toxicity experiments it is therefore important to test the entire endophyte-grass matrix. To give greater confidence in the safety of the AR37 endophyte, a 90-day feeding study was conducted. Endophyte-infected seed was incorporated into the diet of mice at three different dose rates and a comprehensive range of parameters measured over the 90-day period. At the end of the trial a blood sample was taken from each mouse for analysis and tissues and organs collected for histology. As a comparison, pure lolitrem B was also fed to mice at three different dose rates. Including the appropriate control groups, the total number of mice used in the study was 160.

Another potential concern regarding endophyte-expressed compounds is that, since they are expressed in milk, it is highly likely that they are passed from mother to offspring via breast milk. In the risk assessment process children are considered to be part of the vulnerable population as it is possible that they could be particularly susceptible to contaminants [31]. To address this concern a reproduction study was conducted such that mice were fed AR37 or lolitrem B for two complete generations. The dose rates of AR37 and lolitrem B chosen for this study were the high doses used in the feeding study. Litter numbers, gestation periods and the growth of offspring were measured.

These studies have generated the first data set on the safety of lolitrem B and the AR37 endophyte. Consideration of the quantities of toxins found in sheep fat and cow’s milk, along with the high dose rates fed to mice in this study, suggests that neither lolitrem B nor the AR37 endophyte pose a risk to human health.

## 2. Materials and Methods

### 2.1. Animals

Swiss albino mice, bred at AgResearch (Hamilton, New Zealand) were used for both the 90-day feeding study and the two-generation reproduction study. Animals were housed in a temperature-controlled room with a 12 h light-dark cycle and with free access to treatment diets and water. All animal manipulations were approved by the Ruakura Animal Ethics Committee established under the Animal Protection (code of ethical conduct) Regulations Act, 1987 New Zealand) (Approval No. 10916).

### 2.2. Diet Preparation

Diets were based on Teklad Global 2016 mouse food (Harlan UK, Bicester, England), ground to a fine flour using a Udy cyclone sample mill (Udy Corporation, Fort Collins, CO, USA). Pure lolitrem B, available from previous work [8], was added to give concentrations of 1.06, 0.53 or 0.265 ppm for the high, medium and low dose treatment groups, respectively. AR37 endophyte-infected seed (GA66) and seed containing no endophyte (GN139, line N2115) were provided by PGG Wrightson Seeds (Christchurch, New Zealand) and were ground in the same way. These treatment diets contained a total of 30% seed and 70% ground mouse food. To generate the high, medium and low AR37 treatment diets 30, 15 and 7.5% AR37 seed was added to the ground mouse food along with 0, 15 and 22.5% nil endophyte seed, respectively. This yielded epoxyjanthitrem concentrations of 20.1, 10.1 and 5 ppm for high, medium and low diets, respectively. Control diet (ground mouse food with no additions) and nil endophyte treatment diet (ground mouse food to which 30% seed containing no endophyte) were also prepared. 

Diets were prepared in bulk by mixing 300 g of the appropriate dry ingredients (ground mouse food, pure lolitrem B, AR37 endophyte-infected ground seed or nil endophyte seed) in a cake mixer. Aliquots of 100 g were then taken, and water added to form a paste (90 mL for control and lolitrem B diets and 95 mL for seed containing diets). This paste was divided into 15 evenly sized portions and cookie-shaped discs prepared which were then dried in a fan oven (40 °C, 24 h). This resulted in diets with an average moisture content of 15.5, 14.4, 14.7, 12.4, 17.4, 16.9, 17.4 and 17.9% for control, high lolitrem B, medium lolitrem B, low lolitrem B, nil endophyte control, high AR37, medium AR37 and low AR37 treatment diets, respectively. Diets were prepared twice a week during the experimental periods. To check for homogeneity and stability three samples from different areas of a lolitrem B cookie and an AR37 cookie were taken. Each of the three samples were ground in a mortar and pestle before being analyzed by HPLC as described in Section 2.3 below.

### 2.3. Analysis of Lolitrem B and Epoxyjanthitrems

Cookie samples were ground using a mortar and pestle and AR37 seed was ground through a 1 mm screen prior to analysis.

Lolitrem B was extracted from the diet cookie (50 mg) with dichloromethane-methanol (9:1, 1 mL) using an over-over mixer (30 rotations/minute) for 1 h. The extract was then centrifuged (1 min, 5600 *g*, Eppendorf, Hamburg, Germany) and analyzed by HPLC. A standard of pure lolitrem B, available from previous work, was used for quantification [8]. A 4.6 × 250 mm Luna silica column (Phenomenex, Torrance, CA, USA) was utilized with acetonitrile-dichloromethane as eluent (1:4, 1.8 mL/min). Detection used an Agilent Series 1100 fluorescence detector (Agilent Technologies, Waldbronn, Germany) with excitation at 268 nm and emission detection at 440 nm.

Epoxyjanthitrems were extracted from the diet cookie or ground seed (50 mg) with acetone-water (4:1, 1 mL) using the mixer and centrifuge described above before being analyzed by HPLC. Quantification was achieved by comparison with an epoxyjanthitrem I standard which had previously been shown to be at least 95% pure by nuclear resonance spectroscopy. Analysis used a 4.6 × 250 mm C18 column (Phenomenex, Torrance, CA, USA) and detection with an Agilent Series 2200 fluorescence detector (excitation at 333 nm, emission detection at 385 nm).

### 2.4. 90-Day Feeding Study

Due to the large number of mice used in the study (160 in total) the start times of the experiment had to be staggered. Three different start times were used but mice of the same age (4–5 weeks old) were used for each of the three batches.

Mice were randomly allocated to their treatment groups three days before the experiment. All mice were fed control diet for this 3-day period so that they got accustomed to the new form of their diet by the time the experiment started. Each cage group consisted of five mice and each treatment group consisted of two cages (five mice in each) of female mice and two cages (five mice in each) of male mice. Treatment groups were; Group 1—control (cookies containing only ground mouse food), Group 2—low dose lolitrem B (lol B low, 0.265 ppm), Group 3—medium dose lolitrem B (lol B med, 0.53 ppm), Group 4—high dose lolitrem B (lol B high, 1.06 ppm), Group 5—nil endophyte control (nil, 30% endophyte-free seed), Group 6—low dose AR37 (AR37 low, 7.5% AR37, 22.5% nil seed), Group 7—medium dose AR37 (AR37 med, 15% AR37, 15% nil seed) and Group 8—high dose AR37 (AR37 high, 30% AR37 seed). Food consumption of each cage group was measured daily and at the same time the appearance and movement of the mice were observed to check for any changes. The bodyweight of each mouse was measured bi-weekly. By combining the food intake and bodyweight data the dose rate of lolitrem B and epoxyjanthitrems ingested by the mice was calculated for each treatment group (Table 1).

Blood pressure and heart rate were measured on days 0, 21, 42, 63 and 84 on conscious mice using a BP-2000 blood pressure analysis system (Visitech Systems, Apex, NC, USA). For each blood pressure determination 10 measurements were made and averaged. On the same days the motor function and coordination of each mouse was measured using an accelerating rotarod (Rotamex 4 rotarod, Columbus Instruments, Columbus, OH, USA). The rotational speed was increased from 13 to 79 rpm over 12 min and the mean duration to fall (seconds) of two trials measured and averaged. At the conclusion of the 90-day feeding period each mouse was killed using carbon dioxide and a blood sample collected from each animal by heart puncture using heparin as an anti-coagulant. Whole blood was used to measure hematocrit values (HCT), hemoglobin levels (HB), mean corpuscular volumes (MCV), mean corpuscular hemoglobin (MCH), mean corpuscular hemoglobin concentration (MCHC), and red and white blood cell counts. Plasma was then analyzed to determine activities of alanine aminotransferase (ALT), and for concentrations of total protein (TP), albumin (ALB), globulin, sodium, potassium, chloride and creatinine (CRN) (New Zealand Veterinary Pathology, Hamilton, New Zealand). Necropsies were performed to detect macroscopic changes and the weights of brain, heart, kidneys, liver and spleen measured and expressed as percentage of bodyweight. These organs along with the adrenal glands, lungs, pancreas, gastrocnemius, jejunum (3 mm section), ovary/uterus or testes, spinal cord (3 × 2 mm sections), stomach (washed), thymus and urinary bladder were placed in 4% buffered formaldehyde for fixation before being routinely processed for histology. The same pathologist examined all the samples and was blinded to the treatment groups.

### 2.5. Two-Generation Reproduction Study

Four treatment groups, each consisting of five breeding pairs, were used in the study. These were: Group 1—control, Group 2—lolitrem B (1.06 ppm), Group 3—nil endophyte control (30% endophyte-free seed) and Group 4—AR37 (30% AR37 endophyte-infected seed). Five breeding pairs of Swiss mice were mated such that pups were born within days of each other. At weaning (3 weeks old), one male and one female mouse were taken from each of the five litters and allocated to one of the four treatment groups. To allow the originating litters to be identified the mice were distinguished by ear markings. The five male and female mice of each treatment were caged together until they had reached sexual maturity (8 weeks old) after which time the male and female originating from the same litter were paired (brother/sister mating). This yielded five breeding pairs for each treatment group. Daily checks were made for vaginal plugs and 15 days after they were observed, the male mice were removed to prevent post-partum mating. For each litter, the day the pups were born was recorded and gestational period calculated. After two days the pups of each litter were counted, sexed and weighed (all together). To normalize the litter sizes four male and four female mice were left with each mother and the combined weight of the eight pups was measured weekly. For each treatment group there were five litters and after three weeks one male and one female were taken from each of those litters to yield five male and five female mice on each treatment diet. All mice fed on the same treatment diet were housed together but as before mice were marked to allow brothers and sisters from the same litter to be later identified. Each individual mouse was weighed weekly until sexual maturity (8 weeks) after which time the brothers and sisters were paired and mated. The process described above was followed to yield gestational periods, number of pups born and the weight of the litters for this second generation of mice. The litter sizes were again normalized, and the litter weight recorded once a week until weaning. Once again one male and one female mouse were retained from each litter to yield five male and five female mice per treatment group. These mice were weighed once a week until they were 8 weeks of age.

### 2.6. Statistical Analysis

The bodyweight, motor coordination, grip strength, blood pressure and heart rate data were analyzed using repeated measures formulated as linear mixed effects models and fitted using residual maximum likelihood (REML). The random model comprised of random effects for replicate (i.e., the box in the housing arrangement) and mouse. The basic fixed model comprised of effects for treatment group, day, gender and all two- and three-way interactions. Motor coordination was log transformed to stabilize the variance. In the analyses of bodyweight, pre-treatment (i.e., day 0) bodyweight was also included as a fixed effect covariate. Repeated measures on the same mouse over time were assumed to be correlated with a first-order autoregressive structure. The hematological, serum biochemical and organ weight data were analyzed using linear mixed effects models fitted using REML with random effects for replicate (Box) and fixed effects for treatment group, gender and the treatment group by gender interaction. Serum ALT was log transformed to stabilize the variance.

For the reproduction study gestation period, number of pups and litter weights at 1, 2, and 3 weeks were analyzed using ANOVA with treatment, generation and the treatment and generation interaction effects. For individual mouse weight at 3, 4, 5, 6, 7 and 8 weeks of age ANOVA was used with treatment, generation, gender, and all two-way and three-way interaction. In all analyses, the variance components were constrained to be positive, residual diagnostic plots were inspected for evidence of departures from the residual assumptions of normality and constant variance, a 5% significance level was used when assessing the fixed effects, and Fisher’s unprotected least significant differences at the 5% level (LSD (5%)) were used to compare means. Statistical analyses were performed using R version 4.1.1 (2021-08-10) [32].

## 3. Results

### 3.1. Analysis of Treatment Diets

To check homogeneity and stability of the test materials in the treatment diets three samples were taken from different areas of a lolitrem B cookie and an AR37 cookie. These samples, along with a sample of ground AR37 seed, were analyzed by HPLC. Results of the cookie samples were compared to each of the theoretical values which showed that 94, 92 and 97% (average of 94%) and 94, 90 and 89% (average 91%) of lolitrem B and epoxyjanthitrems were recovered from the samples, respectively. This demonstrated that both lolitrem B and the AR37 seed were homogeneously distributed in the treatment diets and that both lolitrem B and the epoxyjanthitrems were stable. As expected, analysis of the AR37 endophyte-infected perennial ryegrass seed showed it to contain all five epoxyjanthitrem compounds, with a total of 70.2 ppm.

### 3.2. 90-Day Feeding Study

#### 3.2.1. Clinical Observations and Appearance

All mice had normal movement, behavior and appearance throughout the entire 90-day experimental period.

#### 3.2.2. Bodyweight and Food Consumption

**Figure 1:**
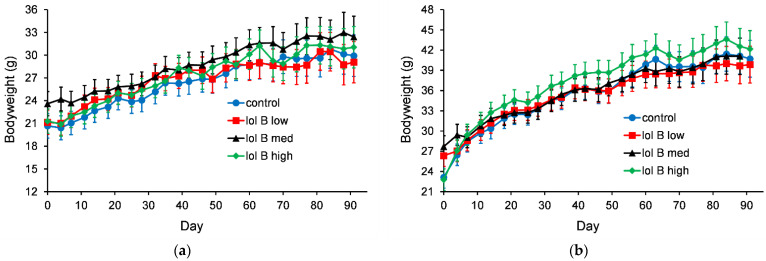
Bodyweight of female mice (**a**) or male mice (**b**) fed diet containing lolitrem B (0, 0.265, 0.53 or 1.06 ppm for control, low, med and high, respectively). The error bars are the standard error obtained from the fitted model.

The bodyweight data showed that the mice gained weight irrespective of the treatment diets. For mice fed diets containing lolitrem B (lol B) the bodyweights of the female mice were closely grouped (Figure 1a). For male mice there were no statistically significant (*p* > 0.05) differences in the 90-day bodyweight data (Figure 1b).

**Figure 2:**
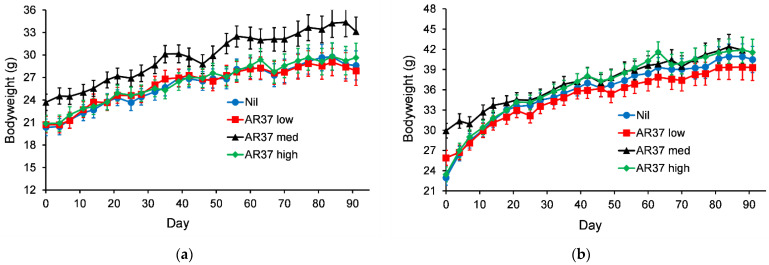
Bodyweight of female mice (**a**) or male mice (**b**) fed a diet containing AR37 endophyte-infected seed (0, 7.5, 15 or 30% for nil, low, med and high, respectively). The error bars are the standard error obtained from the fitted model.

For mice fed AR37 containing diets it was the males that showed a tight grouping (Figure 2b). The female mice fed AR37 med had higher bodyweights (Figure 2a). However, this group had a higher starting bodyweight (day 0) with this difference being maintained throughout the 90-day period.

The food consumption for each cage group relative to their combined bodyweights was calculated which allowed food consumption to be expressed as the gram of food consumed per gram of mouse bodyweight. This showed no differences between the treatment groups. This is consistent with the observation that all mice grew in a similar manner.

#### 3.2.3. Motor Coordination

**Figure 3:**
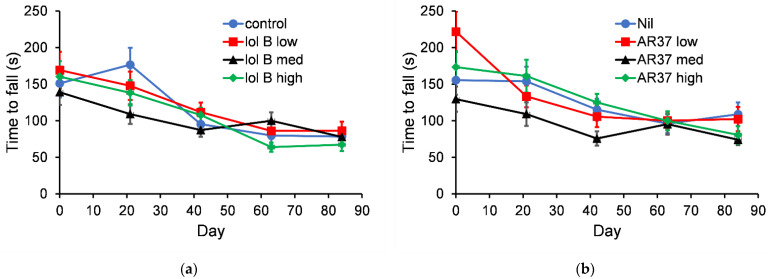
Temporal trend in motor coordination (time to fall) of mice fed (**a**) lolitrem B (0, 0.265, 0.53 or 1.06 ppm for control, low, med and high, respectively) or (**b**) AR37 endophyte-infected seed (0, 7.5, 15 or 30% for nil, low, med and high, respectively). The error bars are the standard error obtained from the fitted model.

Statistical analysis of the rotarod scores showed no evidence of an interaction between gender and treatment (gender.treatment, *p* = 0.79). A graph could therefore be created for the temporal treatment effect pooled over gender (Figure 3). For mice fed diets containing lolitrem B the only statistically significant difference (*p* < 0.05) was between mice fed lol B high and mice fed the other two lolitrem B treatments on day 63. For mice fed diets containing AR37, the only statistically significant difference (*p* < 0.05) was at day 0 between mice fed an AR37 low diet and mice fed an AR37 med diet. 

#### 3.2.4. Heart Rate and Blood Pressure

The heart rates of all mice were measured at the start of the experiment and then every 3 weeks. No consistent differences in the heart rates of groups of mice fed different doses of lolitrem B were observed. For female mice, the greatest variation was observed at day 0 with the only other statistically significant difference (*p* < 0.05) being at day 21 where the mice of the lol B high group had lower heart rates than the mice of the other lol B treatments. However, there was no difference (*p* > 0.05) between the heart rates of mice fed lol B high and those fed the control diet (Table 2). For male mice, the heart rates of the lol B high and control treatments were grouped together. In contrast, the heart rates of the lol B med mice were significantly (*p* < 0.05) higher than those fed other treatments on days 21, 42 and 63 (Table 2).

The heart rates of female mice fed AR37 endophyte-infected seed showed no statistically significant differences (Table 2). For male mice, there was more variability but the heart rates of the AR37 high and nil mice were very similar whereas the heart rates of the AR37 med and AR37 low mice were generally higher, including at day 0 (Table 2).

The systolic and diastolic blood pressures were determined for each mouse at day 0 and then at 3-week intervals. For female mice fed lolitrem B there was a statistically significant difference (*p* < 0.05) between the systolic blood pressure of mice fed a control diet and those fed lol B low/lol B med, lol B low and lol B med on days 42, 63 and 84, respectively (Table 3). Additionally, there was a statistically significant difference between mice fed a lol B low and lol B high diet on day 63 and between mice fed a lol B med and lol B high diet on day 84. For male mice, other than a difference on day 0, the only statistically significant difference (*p* < 0.05) in systolic blood pressure was between mice fed AR37 low and AR37 med on day 84 (Table 3).

For female mice fed an AR37 diet the systolic blood pressures were variable on day 0. The only other statistically significant difference (*p* < 0.05) was on day 42 between mice fed an AR37 low and an AR37 high diet (Table 3). For male mice, other than a difference on day 0, the only statistically significant difference (*p* < 0.05) in systolic blood pressure was between mice fed AR37 low and AR37 med on day 63 (Table 3).

For female mice fed a lol B containing diet the only statistically significant difference (*p* < 0.05) in diastolic blood pressure was between mice fed a control diet and mice fed a lol B low diet on day 63 (Table 4). There were no statistically significant differences in male mice fed lol B.

For female mice fed an AR37 diet there were statistically significant differences on day 21 between mice on the AR37 high diet and the AR37 low diet and on day 63 between mice of the AR37 high treatment group and those on the other AR37 treatment diets (Table 4). For male mice, diastolic blood pressure showed statistically significant differences (*p* < 0.05) between mice fed AR37 low and AR37 med treatments on days 21, 63 and 84 (Table 4). However, on 1/3 of these days the diastolic blood pressures of mice fed AR37 med were higher than those of mice fed AR37 low but for the other 2/3 days the diastolic blood pressure of mice fed AR37 med were lower.

Due to the lack of consistent differences in blood pressure throughout the 90-day period, the lack of dose dependency and the lack of consistent differences between genders, it is unlikely that there was any effect of either lolitrem B or AR37 endophyte-infected seed on systolic or diastolic blood pressures.

#### 3.2.5. Hematological Data

The hematological and serum biochemical data for the samples collected on day 90 are presented in Table 5 and Table 6. For male mice fed diets containing AR37 the only parameter showing a statistically significant difference (*p* < 0.05) was the white blood cell count (Table 5). However, this is unlikely to be of toxicological significance, since although the AR37 low and AR37 med WBC were statistically different (*p* < 0.05) to that of the AR37 high treatment, there were no statistical differences (*p* > 0.05) between the WBC of any of the AR37 treatments and the nil control group. Despite not being statistically significant (*p* > 0.05) in male mice RBC, MCV, MCH and the % eosinophils were statistically different (*p* < 0.05) in female mice (Table 5). However, in every case the values of the AR37 high treatment group were not statistically different (*p* < 0.05) from the nil control demonstrating that the effects noted were not dose dependent. For this reason, as well as the fact that the effects on RBC, MCV, MCH and % eosinophils were gender specific, the observed differences in female mice are unlikely to be toxicologically significant.

For mice fed diets containing lolitrem B it was only the MCV in female mice that showed a statistically significant difference (*p* < 0.05) (Table 6). However, it was only the lol B low treatment group which showed a difference to the control group.

#### 3.2.6. Blood Chemistry Results

The blood chemistry results of the samples from mice fed AR37 containing diets collected on day 90 are presented in Table 7. This shows statistically significant differences (*p* < 0.05) for many of the parameters measured. However, on closer inspection the only parameters that showed a statistical difference (*p* < 0.05) between the AR37 high and the nil endophyte control groups were potassium in male mice and ALT in female mice. The concentration of potassium in the blood of male mice fed the AR37 low and AR37 high treatments were significantly lower than that in the blood of mice fed the nil endophyte control. However, there was no difference between the AR37 med values and the control. Furthermore, although not statistically significant, the potassium concentrations of female mice showed the opposite trend with the AR37 high treatment group having the highest potassium concentration. The ALT levels in female mice showed a statistically significant difference (*p* < 0.05) between those of the nil endophyte control and those of the AR37 high treatments. The AR37 low and med treatment groups did not show this effect and there was no statistical difference (*p* > 0.05) between any of the AR37 treatment groups. There were no statistical differences (*p* > 0.05) observed in the ALT concentrations in male mice. 

Looking at the blood analysis data from mice fed lolitrem B containing diets (Table 8) in the same manner showed that, although a number of statistically significant differences were observed, the only statistically significant difference between the lol B high and control group was in the ALB concentrations in the blood of male mice. In fact, both the lol B med and lol B low dose treatments also showed a statistically significant difference (*p* < 0.05) to the control group. However, for female mice there was no statistically significance difference between the ALB of the three different lolitrem B dose rates and it was only lol B low that was statistically different from the control group.

#### 3.2.7. Organ Weights

The weights of the major organs, measured on day 90, were expressed as the percentage of bodyweight (Table 9). For mice fed a diet containing AR37 there was a statistically significant difference (*p* < 0.05) between the brain weight of mice fed AR37 med and the brain weights of mice fed any of the other treatments. The statistically significant differences (*p* < 0.05) observed in the liver and spleen weights of male mice also looked to have little relevance as in each case the organ weights of the different AR37 treatment groups were not statistically significantly different (*p* > 0.05) from those of the nil control group. A difference was seen between the spleen weight of the female mice fed AR37 med diet and those fed a nil control diet, but no difference was seen between the AR37 high and nil control spleen weights. The weights of the kidneys of male mice were more interesting with a trend of lower kidney weight with higher AR37 consumption although it was only the kidneys of the mice fed AR37 high that were different to the kidney weights of the control mice. However, no statistically significant differences (*p* > 0.05) were noted in the kidney weights of the female mice.

There were no statistically significant differences (*p* > 0.05) between any of the organ weights when expressed as a percentage of bodyweight for mice fed a diet containing lolitrem B (Table 9). 

#### 3.2.8. Histological Examination

Tissues examined from the mice of all treatment groups were within normal histological limits and no lesions were observed within tissues from any of the mice in this study. 

#### 3.2.9. Results Summary

This experiment showed no treatment effect of lolitrem B or the AR37 endophyte on mice. Although some of the parameters measured showed statistically significant differences, they were either only observed in one gender or they showed no dose dependency. For these reasons there was no convincing evidence of any treatment effect and it is unlikely that any of the observed differences are toxicologically significant. The highest dose rates given to mice (0.151 and 3.17 ppm/day for lolitrem B and the epoxyjanthitrems, respectively) did not cause any toxicologically significant effects. It is also important to note that the mice fed AR37 endophyte-infected seed were ingesting the entire seed matrix. Therefore, in addition to epoxyjanthitrems, mice would also have been exposed to the myriad of other compounds produced by the AR37 endophyte.

#### 3.2.10. Implications

To determine whether these toxins could pose a threat to human health the dose rates given to mice can be compared to those which could be ingested by a human eating animal products. An experiment whereby cows were fed either common-toxic (expressing lolitrem B) or AR37 (expressing epoxyjanthitrems) endophyte-infected herbage for 12 days demonstrated residues of these toxins in milk [26]. Analysis showed maximum concentrations of 5 ppb lolitrem B and 109 ppb epoxyjanthitrems in the milk of cows fed common-toxic or AR37 endophyte-infected ryegrass, respectively. To reach the same daily dose rate as that fed to the mice in our study a 70 kg human would have to drink just over 2000 litres of milk per day. When using animal data in a human risk assessment a 10-times safety factor is applied to account for the species difference and a further 10-times safety factor is applied to account for the fact that some of the human population, such as the young or the elderly, may be more susceptible to contaminants [31]. Applying this combined 100-fold safety factor, a 70 kg human would still have to drink 20 litres of milk/day from cows grazing endophyte-infected pastures to yield a dose rate equivalent to that which we fed to mice with no adverse effects.

Residues of lolitrem B and epoxyjanthitrems have also been detected in the fat of sheep grazing common-toxic and AR37 endophyte-infected pastures, respectively. An experiment was conducted whereby monthly fat biopsies were taken from sheep grazing endophyte-infected pastures over a 6-month period [27]. The maximum concentration of lolitrem B in the fat of sheep grazing common-toxic endophyte-infected pastures was 62 ppb and that of epoxyjanthitrems in the fat of sheep grazing AR37 endophyte-infected pastures was 1032 ppb. To reach the daily dose rate fed to mice in the current study a 70 kg human would have to consume 170 kg and 215 kg of fat/day from sheep grazing common-toxic and AR37 endophyte-infected pastures, respectively. If we assume meat products contain 20% fat then this would translate into 8500 and 11,000 kg of meat/day from sheep grazing common-toxic and AR37 endophyte-infected pastures, respectively. Applying the 100-fold safety factor to these figures results in values of 8.5 and 11 kg of meat/day.

A risk assessment requires two pieces of information; how toxic is the compound and what is the maximum amount a human could consume. Regarding toxicity, mice in our study showed no adverse effects, even at the highest dose rate which means that toxicity is greater than 0.151 or 3.17 ppm/day for lolitrem B or epoxyjanthitrems, respectively. Regarding the latter question, we need to consider whether toxin residues could exceed that reported in the milk and fat of animals ingesting endophyte-infected pasture. Both the milk and fat studies were conducted when the toxin concentrations in endophyte-infected herbage would have been at their highest (summer months). Furthermore, ryegrass staggers, the animal disease induced by these toxins was sufficiently severe that removal of animals from their treatments was necessary. This suggests that animals could not consume greater quantities of lolitrem B or epoxyjanthitrems and as such, the concentrations of these toxins reported in milk could be considered the highest possible. Rather than accumulation of lolitrem B or epoxyjanthitrems in sheep fat it was demonstrated that concentrations responded quickly to that being ingested by the animal [27]. The toxin residues reported in fat are therefore unlikely to be exceeded.

Although the concentrations of lolitrem B and epoxyjanthitrems have not been tracked through the manufacture of foods derived from animal products it appears highly unlikely that a consumer could ingest anywhere near the quantities of fat or milk required to present a risk to human health. This assumption incorporates the 100-fold safety factor required to consider animal data in the assessment of human health risk.

### 3.3. Two-Generation Reproduction Study

#### 3.3.1. Gestation Period and Number of Pups Born per Litter

The gestation period and the number of pups born by mice fed lolitrem B (1.06 ppm) and AR37 endophyte-infected seed (30%) for two generations are presented in Table 10. There was a statistically significant difference (*p* < 0.05) between the gestation period of mice fed a control diet and a lolitrem B diet for the 1st but not the 2nd generation of mice. There were no statistically significant differences (*p* > 0.05) in the number of pups born by mice fed any of the treatment groups in either generation.

#### 3.3.2. Growth of Pups from Birth to 3 Weeks of Age

**Figure 4:**
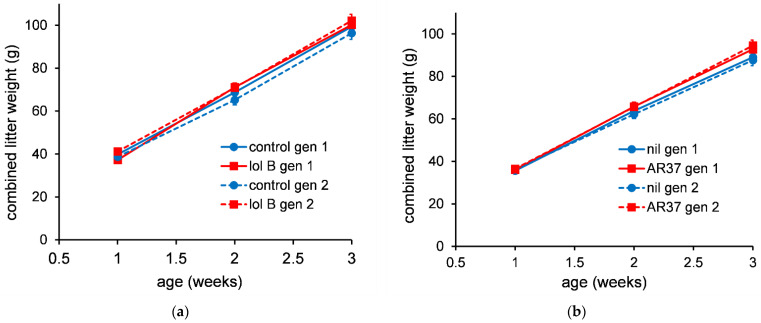
The combined litter weights (*n* = 5) for two generations fed: (**a**) control or diet containing lolitrem B (1.06 ppm); (**b**) diet containing nil or AR37 endophyte-infected seed (30% seed). The error bars are the standard error obtained from the fitted model.

After the litters were born 4 male and 4 female mice were left with their mother and the combined weight of these 8 pups were measured once a week until they were three weeks old and ready for weaning (Figure 4). This same procedure was followed for the 2nd generation of offspring. There were no statistically significant differences (*p* > 0.05) in litter weights between mice fed control and lolitrem B diets or between nil and AR37 endophyte-infected seed in either generation of mice. 

#### 3.3.3. Growth of Pups from 3 to 8 Weeks of Age

**Figure 5:**
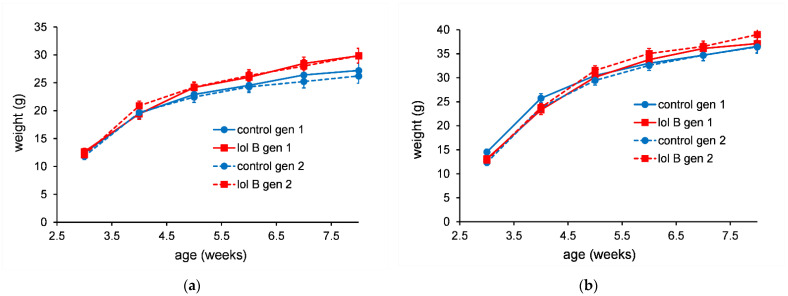
The average weight of individual female mice (**a**) or male mice (**b**) (*n* = 5) fed diet containing lolitrem B (1.06 ppm). The error bars are the standard error obtained from the fitted model.

**Figure 6:**
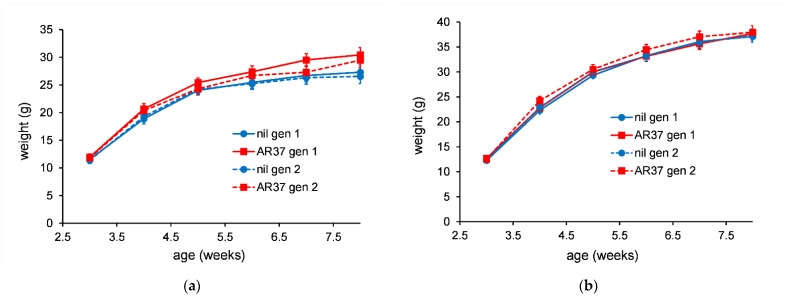
The average weight of individual female mice (**a**) or male mice (**b**) (*n* = 5) fed diet containing AR37 endophyte-infected seed (30%). The error bars are the standard error obtained from the fitted model.

Individual bodyweights were measured for all mice fed the different treatments. This was done for two generations of mice. For both male and female mice there were no statistically significant differences (*p* > 0.05) between mice fed control or lolitrem B diets (Figure 5) or for mice fed diets containing nil or AR37 endophyte-infected seed (30%) (Figure 6) in either generation 1 or 2.

#### 3.3.4. Results Summary

Since lolitrem B and epoxyjanthitrem residues have been detected in milk it is possible that if a mother consumed animal products containing these toxins they could be passed from the mother to offspring via the placenta or breast milk. To test whether this could be problematic a study was conducted whereby two generations of mice were raised on treatment diets. Treatment diets contained either 1.06 ppm lolitrem B or 30% AR37 endophyte-infected seed (20.1 ppm epoxyjanthitrems). No effect was observed on the gestation period, number of pups born or the growth of pups either pre-weaning or post-weaning in the reproduction study. As detailed earlier, mice fed lolitrem B or AR37 endophyte-infected seed in this experiment would have received a dose rate far higher than that which could be achieved by a human eating contaminated food. 

## 4. Conclusions

These experiments are the first to assess lolitrem B and the epoxyjanthitrem producing endophyte, AR37 for potential toxicity to mammals. Dose rates of lolitrem B and the AR37 endophyte, far higher than those which could be ingested by the consumption of animal products by humans, were not associated with any toxicity in mice. By considering the dose rates fed to mice and the concentrations reported in animal products neither lolitrem B nor the AR37 endophyte raise any concern for human health. However, a species difference in toxin metabolism cannot be excluded.

Additional novel endophytes are constantly being commercialized and each endophyte-grass association will produce a different profile of secondary metabolites. Although the animal safety of new endophytes is currently comprehensively tested consideration of food safety and human health is also required.

## Figures and Tables

**Table 1:** Dose rate of lolitrem B or epoxyjanthitrems (mg/kg/day) consumed for 90 days.

Gender	Lol B Low	Lol B Med	Lol B High	AR37 Low EJ	AR37 Med EJ	AR37 High EJ
**Female**	0.040	0.079	0.171	0.810	1.55	3.44
**Male**	0.036	0.068	0.151	0.786	1.43	3.17

**Table 2:** Heart rate of mice fed a diet containing lolitrem B (0, 0.265, 0.53 or 1.06 ppm for control, low, med and high, respectively) or AR37 endophyte-infected seed diet (0, 7.5, 15 or 30% for nil, low, med and high, respectively).

	Nil	AR37 Low	AR37 Med	AR37 High	Control	Lol B Low	Lol B Med	Lol B High
***Females***								
0	655 ± 15	649 ± 15	621 ± 15	638 ± 15	650 ± 15 ^abc^	685 ± 15 ^a^	660 ± 15 ^ab^	613 ± 15 ^c^
21	645 ± 15	673 ± 15	653 ± 15	638 ± 15	658 ± 15 ^ab^	673 ± 15 ^a^	675 ± 15 ^a^	628 ± 15 ^b^
42	666 ± 15	662 ± 15	653 ± 15	678 ± 15	660 ± 15	663 ± 15	652 ± 15	625 ± 15
63	664 ± 15	660 ± 15	653 ± 15	671 ± 15	654 ± 15	667 ± 15	662 ± 15	651 ± 15
84	647 ± 15	684 ± 15	667 ± 15	661 ± 15	651 ± 15	678 ± 15	646 ± 15	641 ± 15
***Males***								
0	637 ± 15 ^ab^	672 ± 15 ^a^	672 ± 15 ^a^	613 ± 15 ^b^	620 ± 15 ^c^	675 ± 15 ^a^	649 ± 15 ^abc^	624 ± 15 ^bc^
21	617 ± 15 ^c^	672 ± 15 ^ab^	685 ± 15 ^a^	638 ± 15 ^bc^	626 ± 15 ^bc^	662 ± 15 ^ab^	683 ± 15 ^a^	615 ± 15 ^c^
42	648 ± 15 ^b^	678 ± 15 ^ab^	704 ± 15 ^a^	643 ± 15 ^b^	664 ± 15 ^ab^	666 ± 15 ^ab^	696 ± 15 ^a^	647 ± 15 ^b^
63	646 ± 15 ^b^	662 ± 15 ^ab^	690 ± 15 ^a^	659 ± 15 ^ab^	635 ± 15 ^b^	660 ± 15 ^b^	705 ± 15 ^a^	638 ± 15 ^b^
84	647 ± 15	673 ± 15	672 ± 15	666 ± 15	655 ± 15	645 ± 15	638 ± 15	668 ± 15

Values are means ± standard error (*n* = 10). Fisher’s least significant difference was used to compare treatment means. Two means that have no letter in common are statistically different at the 5% level. If no letters are present, no statistical difference was found between the groups.

**Table 3:** Systolic blood pressure (mm/Hg) of mice fed a diet containing lolitrem B (0, 0.265, 0.53 or 1.06 ppm for control, low, med and high, respectively) or AR37 endophyte-infected seed diet (0, 7.5, 15 or 30% for nil, low, med and high, respectively).

	Nil	AR37 Low	AR37 Med	AR37 High	Control	Lol B Low	Lol B Med	Lol B High
***Females***								
0	104 ± 3 ^b^	112 ± 3 ^ab^	117 ± 3 ^a^	106 ± 3 ^b^	112 ± 3	114 ± 3	112 ± 3	109 ± 3
21	113 ± 3	116 ± 3	116 ± 3	117 ± 3	123 ± 3	117 ± 3	115 ± 3	115 ± 3
42	120 ± 3 ^ab^	110 ± 3 ^c^	118 ± 3 ^abc^	123 ± 3 ^a^	123 ± 3 ^a^	115 ± 3 ^bc^	111 ± 3 ^c^	119 ± 3 ^abc^
63	115 ± 3	115 ± 3	117 ± 3	122 ± 3	119 ± 3 ^a^	107 ± 3 ^b^	113 ± 3 ^ab^	117 ± 3 ^a^
84	117 ± 3	116 ± 3	110 ± 3	116 ± 3	123 ± 3 ^a^	116 ± 3 ^ab^	108 ± 3 ^c^	115 ± 3 ^bc^
***Males***								
0	98 ± 3 ^b^	110 ± 3 ^a^	114 ± 3 ^a^	101 ± 3 ^b^	99 ± 3 ^c^	110 ± 3 ^a^	108 ± 3 ^ab^	100 ± 3 ^bc^
21	113 ± 3	115 ± 3	118 ± 3	117 ± 3	112 ± 3	116 ± 3	116 ± 3	116 ± 3
42	120 ± 3	117 ± 3	122 ± 3	114 ± 3	121 ± 3	119 ± 3	118 ± 3	122 ± 3
63	117 ± 3 ^ab^	122 ± 3 ^a^	112 ± 3 ^b^	117 ± 3 ^ab^	122 ± 3	120 ± 3	118 ± 3	116 ± 3
84	118 ± 3	123 ± 3	117 ± 3	120 ± 3	119 ± 3 ^ab^	126 ± 3 ^a^	116 ± 3 ^b^	123 ± 3 ^ab^

Values are means ± standard error (*n* = 10). Fisher’s least significant difference was used to compare treatment means. Two means that have no letter in common are statistically different at the 5% level. If no letters are present, no statistical difference was found between the groups.

**Table 4:** Diastolic blood pressure (mm/Hg) of mice fed a diet containing lolitrem B (0, 0.265, 0.53 or 1.06 ppm for control, low, med and high, respectively) or AR37 endophyte-infected seed diet (0, 7.5, 15 or 30% for nil, low, med and high, respectively).

	Nil	AR37 Low	AR37 Med	AR37 High	Control	Lol B Low	Lol B Med	Lol B High
***Females***								
0	71 ± 3	74 ± 3	74 ± 3	74 ± 3	76 ± 3	78 ± 3	75 ± 3	76 ± 3
21	77 ± 3 ^ab^	70 ± 3 ^b^	77 ± 3 ^ab^	81 ± 3 ^a^	81 ± 3	77 ± 3	74 ± 3	77 ± 3
42	77 ± 3	70 ± 3	73 ± 3	77 ± 3	76 ± 3	77 ± 3	70 ± 3	75 ± 3
63	74 ± 3 ^b^	75 ± 3 ^b^	73 ± 3 ^b^	85 ± 3 ^a^	79 ± 3 ^a^	70 ± 3 ^b^	73 ± 3 ^ab^	74 ± 3 ^ab^
84	72 ± 3	76 ± 3	71 ± 3	71 ± 3	77 ± 3	70 ± 3	71 ± 3	73 ± 3
***Males***								
0	69 ± 3	76 ± 3	76 ± 3	77 ± 3	73 ± 3	71 ± 3	71 ± 3	79 ± 3
21	81 ± 3 ^ab^	73 ± 3 ^b^	80 ± 3 ^ab^	81 ± 3 ^a^	79 ± 3	74 ± 3	71 ± 3	79 ± 3
42	79 ± 3	76 ± 3	77 ± 3	72 ± 3	76 ± 3	76 ± 3	79 ± 3	80 ± 3
63	75 ± 3 ^bc^	83 ± 3 ^ab^	71 ± 3 ^c^	74 ± 3 ^c^	75 ± 3	75 ± 3	74 ± 3	79 ± 3
84	77 ± 3 ^abc^	80 ± 3 ^a^	70 ± 3 ^c^	79 ± 3 ^ab^	76 ± 3	77 ± 3	79 ± 3	80 ± 3

Values are means ± standard error (*n* = 10). Fisher’s least significant difference was used to compare treatment means. Two means that have no letter in common are statistically different at the 5% level. If no letters are present, no statistical difference was found between the groups.

**Table 5:** Hematology data of mice fed nil, AR37 low, AR37 med or AR37 high diets (containing 0, 7.5, 15 or 30% endophyte-infecteed seed, respectively) for 90 days.

	Nil	AR37 Low	AR37 Med	AR37 High
***Females***				
HCT (L/L)	0.42 ± 0.01	0.44 ± 0.01	0.43 ± 0.01	0.42 ± 0.01
HB (g/L)	143.5 ± 3.3	149.8 ± 3.5	148.2 ± 3.5	144.0 ± 3.3
RBC (×10^12^/L)	9.00 ± 0.22 ^b^	9.84 ± 0.24 ^a^	9.26 ± 0.24 ^ab^	9.05 ± 0.22 ^b^
MCV (fL)	46.9 ± 0.5 ^a^	44.9 ± 0.5 ^b^	46.8 ± 0.5 ^a^	46.5 ± 0.5 ^ab^
MCH (pg)	16.0 ± 0.2 ^a^	15.2 ± 0.2 ^b^	16.1 ± 0.2 ^a^	15.9 ± 0.2 ^ab^
MCHC (g/L)	342.8 ± 3.0	338.6 ± 3.2	342.8 ± 3.0	345.4 ± 3.1
WBC (×10^9^/L)	4.88 ± 0.88	6.20 ± 0.98	6.13 ± 0.88	4.61 ± 0.92
Neutrophil (%)	14.6 ± 4.5	18.3 ± 4.9	15.5 ± 4.5	18.6 ± 4.6
Lymphocyte (%)	76.7 ± 4.7	75.9 ± 5.1	78.3 ± 4.7	74.6 ± 4.9
Monocyte (%)	5.5 ± 0.6 ^a^	3.9 ± 0.6 ^ab^	3.6 ± 0.6 ^b^	4.1 ± 0.6 ^ab^
Eosinophil (%)	3.0 ± 0.6	2.1 ± 0.7	2.9 ± 0.6	3.0 ± 0.6
***Males***				
HCT (L/L)	0.41 ± 0.01	0.42 ± 0.01	0.40 ± 0.01	0.42 ± 0.01
HB (g/L)	135.4 ± 3.3	137.7 ± 3.3	134.0 ± 3.3	138.4.0 ± 3.3
RBC (×10^12^/L)	8.65 ± 0.22	8.85 ± 0.22	8.49 ± 0.22	8.97 ± 0.22
MCV (fL)	47.3 ± 0.5	47.0 ± 0.5	47.3 ± 0.5	46.9 ± 0.5
MCH (pg)	15.6 ± 0.2	15.7 ± 0.2	15.7 ± 0.2	15.6 ± 0.2
MCHC (g/L)	332.0 ± 3.0	331.9 ± 3.0	336.3 ± 3.0	329.5.3 ± 3.0
WBC (×10^9^/L)	5.82 ± 0.88 ^ab^	4.62 ± 0.88 ^b^	3.86 ± 0.88 ^b^	7.65 ± 0.88 ^a^
Neutrophil (%)	27.3 ± 4.5	39.6 ± 4.5	31.1 ± 4.5	40.7 ± 4.5
Lymphocyte (%)	64.8 ± 4.7	55.0 ± 4.7	63.6 ± 4.7	52.3 ± 4.7
Monocyte (%)	4.9 ± 0.6	3.2 ± 0.6	3.3 ± 0.6	4.6 ± 0.6
Eosinophil (%)	3.3 ± 0.6	2.8 ± 0.7	2.5 ± 0.7	2.4 ± 0.6

Values are means ± standard error (*n* = 10). Fisher’s least significant difference was used to compare treatment means. Two means that have no letter in common are statistically different at the 5% level. If no letters are present, no statistical difference was found between the groups.

**Table 6:** Hematology data of mice fed control, lol B low, lol B med or lol B high diets (containing 0, 0.265, 0.53 or 1.06 ppm lolitrem B, respectively) for 90 days.

	Control	Lol B Low	Lol B Med	Lol B High
***Females***				
HCT (L/L)	0.43 ± 0.01	0.45 ± 0.01	0.43 ± 0.01	0.43 ± 0.01
HB (g/L)	146.7 ± 3.0	151.2 ± 3.0	145.6 ± 3.0	146.0 ± 3.0
RBC (×10^12^/L)	9.21 ± 0.20	9.80 ± 0.20	9.15 ± 0.20	9.15 ± 0.20
MCV (fL)	46.8 ± 0.40 ^ab^	45.8 ± 0.40 ^b^	46.8 ± 0.40 ^ab^	47.4 ± 0.40 ^a^
MCH (pg)	15.9 ± 0.17	15.5 ± 0.17	16.0 ± 0.17	16.0 ± 0.17
MCHC (g/L)	340.7 ± 2.1	337.6 ± 2.1	340.3 ± 2.1	337.2 ± 2.1
WBC (×10^9^/L)	5.41 ± 0.71	5.31 ± 0.71	5.96 ± 0.71	5.52 ± 0.71
Neutrophil (%)	22.8 ± 4.3	20.5 ± 4.3	17.1 ± 4.3	18.3 ± 4.3
Lymphocyte (%)	70.8 ± 4.6	74.7 ± 4.6	76.7 ± 4.6	74.5 ± 4.6
Monocyte (%)	4.2 ± 0.6	3.0 ± 0.6	3.9 ± 0.6	4.4 ± 0.6
Eosinophil (%)	2.4 ± 0.7	2.0 ± 0.7	2.4 ± 0.7	3.1 ± 0.7
***Males***				
HCT (L/L)	0.41 ± 0.01	0.42 ± 0.01	0.40 ± 0.01	0.42 ± 0.01
HB (g/L)	135.4 ± 3.0	137.4 ± 3.0	131.3 ± 3.0	135.6 ± 3.0
RBC (×10^12^/L)	8.72 ± 0.20	8.85 ± 0.20	8.34 ± 0.20	8.73 ± 0.20
MCV (fL)	47.3 ± 0.40	47.0 ± 0.40	47.3 ± 0.40	46.9 ± 0.40
MCH (pg)	15.6 ± 0.17	15.5 ± 0.17	15.7 ± 0.17	15.6 ± 0.17
MCHC (g/L)	331.3 ± 2.1	329.1 ± 2.1	330.5 ± 2.1	327.7 ± 2.1
WBC (×10^9^/L)	5.50 ± 0.71	5.27 ± 0.71	6.97 ± 0.71	5.98 ± 0.71
Neutrophil (%)	29.7 ± 4.3	31.9 ± 4.3	34.2 ± 4.3	39.0 ± 4.3
Lymphocyte (%)	63.2 ± 4.6	60.0 ± 4.6	59.8 ± 4.6	53.6 ± 4.6
Monocyte (%)	4.8 ± 0.6	5.1 ± 0.6	4.3 ± 0.6	4.7 ± 0.6
Eosinophil (%)	3.3 ± 0.8	2.0 ± 0.7	2.1 ± 0.7	2.7 ± 0.6

Values are means ± standard error (*n* = 10). Fisher’s least significant difference was used to compare treatment means. Two means that have no letter in common are statistically different at the 5% level. If no letters are present, no statistical difference was found between the groups.

**Table 7:** Blood chemistry of mice fed nil, AR37 low, AR37 med or AR37 high diets (containing 0, 7.5, 15 or 30% endophyte-infected seed, respectively) for 90 days.

Item	Nil	AR37 Low	AR37 Med	AR37 High
***Females***				
Log ALT (IU/L)	3.45 ± 0.13 ^b^	3.51 ± 0.13 ^ab^	3.75 ± 0.13 ^ab^	3.87 ± 0.13 ^a^
TP (g/L)	56.3 ± 0.8 ^a^	53.3 ± 0.8 ^b^	55.6 ± 0.8 ^ab^	56.8 ± 0.8 ^a^
ALB (g/L)	36.7 ± 0.6 ^a^	33.7 ± 0.6 ^b^	35.5 ± 0.6 ^ab^	36.9 ± 0.6 ^a^
Globulin (g/L)	19.6 ± 0.4	19.7 ± 0.4	20.1 ± 0.4	19.9 ± 0.4
CRN (µmol/L)	7.5 ± 0.4 ^a^	7.1 ± 0.4 ^a^	5.7 ± 0.4 ^b^	7.8 ± 0.4 ^a^
A/G	1.89 ± 0.04 ^a^	1.71 ± 0.04 ^b^	1.79 ± 0.04 ^ab^	1.87 ± 0.04 ^a^
Na (mmol/L)	152 ± 0.5 ^bc^	153 ± 0.5 ^ab^	154 ± 0.5 ^a^	150 ± 0.5 ^c^
K (mmol/L)	7.4 ± 0.3	7.0 ± 0.3	7.8 ± 0.3	7.8 ± 0.3
Cl (mmol/L)	112 ± 0.8 ^a^	109 ± 0.9 ^b^	112 ± 0.8 ^ab^	110 ± 0.9 ^ab^
***Males***				
Log ALT (IU/L)	3.44 ± 0.13	3.42 ± 0.13	3.67 ± 0.13	3.66 ± 0.13
TP (g/L)	54.5 ± 0.8 ^ab^	49.6 ± 0.8 ^c^	52.2 ± 0.8 ^b^	55.6 ± 0.8 ^a^
ALB (g/L)	31.2 ± 0.6 ^a^	27.8 ± 0.6 ^b^	30.2 ± 0.6 ^a^	31.4 ± 0.6 ^a^
Globulin (g/L)	23.3 ± 0.4 ^a^	21.8 ± 0.4 ^b^	21.8 ± 0.4 ^b^	24.2 ± 0.4 ^a^
CRN (µmol/L)	6.0 ± 0.4 ^ab^	4.9 ± 0.4 ^b^	5.0 ± 0.4 ^b^	6.9 ± 0.4 ^a^
A/G	1.35 ± 0.04	1.28 ± 0.04	1.38 ± 0.04	1.32 ± 0.04
Na (mmol/L)	151 ± 0.5 ^b^	155 ± 0.5 ^a^	155 ± 0.5 ^a^	151 ± 0.5 ^b^
K (mmol/L)	9.4 ± 0.3 ^a^	7.9 ± 0.3 ^b^	8.7 ± 0.3 ^ab^	8.2 ± 0.3 ^b^
Cl (mmol/L)	109 ± 0.8 ^bc^	110 ± 0.8 ^ab^	113 ± 0.8 ^a^	107 ± 0.8 ^c^

Values are means ± standard error (*n* = 10). Fisher’s least significant difference was used to compare treatment means. Two means that have no letter in common are statistically different at the 5% level. If no letters are present, no statistical difference was found between the groups.

**Table 8:** Blood chemistry of mice fed control, lol B low, lol B med or lol B high diets (containing 0, 0.265, 0.53 or 1.06 ppm lolitrem B, respectively) for 90 days.

Item	Control	Lol B Low	Lol B Med	Lol B High
***Females***				
Log ALT (IU/L)	3.17 ± 0.14	3.46 ± 0.14	3.47 ± 0.14	3.36 ± 0.14
TP (g/L)	58.1 ± 0.9 ^a^	53.3 ± 0.9 ^b^	54.8 ± 0.9 ^b^	58.6 ± 0.9 ^a^
ALB (g/L)	37.7 ± 0.8 ^a^	33.1 ± 0.8 ^b^	35.7 ± 0.8 ^ab^	37.8 ± 0.8 ^a^
Globulin (g/L)	20.4 ± 1.0	20.2 ± 1.0	19.1 ± 1.0	20.8 ± 1.0
CRN (µmol/L)	7.4 ± 0.5 ^a^	6.4 ± 0.5 ^ab^	5.8 ± 0.5 ^b^	7.1 ± 0.5 ^ab^
A/G	1.87 ± 0.06 ^a^	1.65 ± 0.06 ^b^	1.87 ± 0.06 ^a^	1.83 ± 0.06 ^ab^
Na (mmol/L)	151 ± 0.8	153 ± 0.8	153 ± 0.8	152 ± 0.8
K (mmol/L)	6.9 ± 0.4	6.9 ± 0.4	8.0 ± 0.4	7.6 ± 0.4
Cl (mmol/L)	110 ± 0.7	109 ± 0.7	112 ± 0.7	110 ± 0.7
***Males***				
Log ALT (IU/L)	3.40 ± 0.15	3.33 ± 0.15	3.53 ± 0.15	3.58 ± 0.15
TP (g/L)	56.1 ± 0.9 ^a^	52.7 ± 0.9 ^b^	53.8 ± 0.9 ^ab^	53.2 ± 0.9 ^ab^
ALB (g/L)	32.4 ± 0.8 ^a^	27.6 ± 0.8 ^b^	29.5 ± 0.8 ^b^	29.7 ± 0.8 ^b^
Globulin (g/L)	23.7 ± 1.0	25.1 ± 1.0	24.3 ± 1.0	23.5 ± 1.0
CRN (µmol/L)	5.5 ± 0.5	5.2 ± 0.5	4.6 ± 0.5	6.1 ± 0.5
A/G	1.37 ± 0.06 ^a^	1.14 ± 0.06 ^b^	1.29 ± 0.06 ^ab^	1.25 ± 0.06 ^ab^
Na (mmol/L)	151 ± 0.8 ^b^	154 ± 0.8 ^ab^	155 ± 0.8 ^a^	155 ± 0.8 ^b^
K (mmol/L)	8.3 ± 0.4	8.1 ± 0.4	8.1 ± 0.4	8.7 ± 0.4
Cl (mmol/L)	107 ± 0.7 ^b^	108 ± 0.7 ^b^	111 ± 0.7 ^a^	109 ± 0.7 ^b^

Values are means ± standard error (*n* = 10). Fisher’s least significant difference was used to compare treatment means. Two means that have no letter in common are statistically different at the 5% level. If no letters are present, no statistical difference was found between the groups.

**Table 9:** Relative organ weight data (% of total bodyweight) for mice fed AR37 or lolitrem B containing treatment diets for 90 days.

	Brain	Heart	Kidneys	Liver	Spleen
***Females***					
Control	1.79 ± 0.07	0.52 ± 0.03	1.18 ± 0.10	4.16 ± 0.16	0.42 ± 0.03
Lol B low	1.82 ± 0.07	0.55 ± 0.03	1.29 ± 0.10	3.89 ± 0.16	0.41 ± 0.03
Lol B med	1.72 ± 0.07	0.55 ± 0.03	1.20 ± 0.10	3.94 ± 0.16	0.41 ± 0.03
Lol B high	1.74 ± 0.07	0.53 ± 0.03	1.19 ± 0.10	4.23 ± 0.16	0.41 ± 0.03
Nil	1.84 ± 0.04 ^a^	0.57 ± 0.02	1.20 ± 0.08	4.06 ± 0.14	0.41 ± 0.01 ^a^
AR37 low	1.85 ± 0.04 ^a^	0.53 ± 0.02	1.21 ± 0.08	4.04 ± 0.14	0.39 ± 0.01 ^ab^
AR37 med	1.65 ± 0.04 ^b^	0.51 ± 0.02	1.13 ± 0.08	4.11 ± 0.15	0.35 ± 0.01 ^b^
AR37 high	1.80 ± 0.04 ^a^	0.52 ± 0.02	1.13 ± 0.08	4.21 ± 0.14	0.41 ± 0.01 ^a^
***Males***					
Control	1.24 ± 0.07	0.55 ± 0.03	1.76 ± 0.10	4.02 ± 0.16	0.29 ± 0.03
Lol B low	1.30 ± 0.07	0.55 ± 0.03	1.93 ± 0.10	4.27 ± 0.16	0.30 ± 0.03
Lol B med	1.26 ± 0.07	0.51 ± 0.03	1.69 ± 0.10	3.82 ± 0.16	0.27 ± 0.03
Lol B high	1.24 ± 0.07	0.58 ± 0.03	1.87 ± 0.10	4.08 ± 0.16	0.32 ± 0.03
Nil	1.28 ± 0.04	0.56 ± 0.08	2.09 ± 0.08 ^a^	4.00 ± 0.15 ^ab^	0.29 ± 0.01 ^ab^
AR37 low	1.30 ± 0.04	0.55 ± 0.08	2.07 ± 0.08 ^a^	4.34 ± 0.14 ^a^	0.31 ± 0.01 ^ab^
AR37 med	1.31 ± 0.04	0.58 ± 0.08	1.84 ± 0.08 ^ab^	3.84 ± 0.14 ^b^	0.27 ± 0.01 ^b^
AR37 high	1.26 ± 0.04	0.54 ± 0.08	1.67 ± 0.08 ^b^	4.09 ± 0.14 ^ab^	0.34 ± 0.01 ^a^

Values are means ± standard error (*n* = 10). Fisher’s least significant difference was used to compare treatment means. Two means that have no letter in common are statistically different at the 5% level. If no letters are present, no statistical difference was found between the groups.

**Table 10:** Gestation period and pups born/litter for mice fed diets containing lolitrem B (1.06 ppm) or AR37 seed (30%) for 2 generations.

Treatment	Gestation Period (Days)	Number of Pups Born
1st generation control	19.8 ± 0.41 ^a^	11.8 ± 1.00
1st generation lolitrem B	18.6 ± 0.37 ^b^	11.5 ± 1.00
1st generation nil	18.8 ± 0.41	12.8 ± 1.12
1st generation AR37	18.6 ± 0.37	12.6 ± 1.00
2nd generation control	18.8 ± 0.37	11.8 ± 1.00
2nd generation lolitrem B	19.0 ± 0.37	12.8 ± 1.12
2nd generation nil	18.8 ± 0.41	12.6 ± 1.00
2nd generation AR37	19.0 ± 0.37	10.8 ± 1.00

Values are means ± standard error (*n* = 5). Fisher’s least significant difference was used to compare treatment means. Two means that have no letter in common are statistically different at the 5% level. If no letters are given, the overall *p*-value was not statistically different at the 5% level.

## Data Availability

The data presented in this study are available on request from the corresponding author.
